# Patient life aspirations in the context of Duchenne Muscular Dystrophy: a mixed-methods case–control study

**DOI:** 10.1186/s41687-022-00500-8

**Published:** 2022-09-14

**Authors:** Carolyn E. Schwartz, Elijah Biletch, Richard B. B. Stuart, Bruce D. Rapkin

**Affiliations:** 1 DeltaQuest Foundation, Inc., 31 Mitchell Road, Concord, MA 01742 USA; 2 Departments of Medicine and Orthopaedic Surgery, Tufts University Medical School, Boston, MA USA; 3 Department of Epidemiology and Population Health, Albert Einstein College of Medicine, Bronx, NY USA

**Keywords:** Duchenne Muscular Dystrophy, Aspirations, Goals, Patients, Case–control, Mixed methods

## Abstract

**Background:**

Aspirations refer to wishes, ways of defining quality of life (QOL), and life goals. Living with chronic illness likely impacts a person’s life aspirations. Duchenne Muscular Dystrophy (DMD) is an inherited disorder characterized by the inevitable and progressive loss of ambulation and independence. The present cross-sectional case–control study investigated how aspirations differed between people with DMD and a stratified comparison group of nationally representative children/adults.

**Methods:**

A web-based survey was administered October through December 2020. Recruitment was stratified by age group: 8–12, 13–17, and >  = 18, reflecting the DMD disability trajectory. Aspirations were measured using qualitative (open-ended) and quantitative (closed-ended) questions. Qualitative prompts asked participants about wishes, how they would define “QOL”; and goals; answers to the prompts were then coded by six trained raters. Quantitative questions included 29 closed-ended goal-delineation items from the QOL Appraisal Profile_v2_. These data were analyzed using multivariate models adjusting for propensity scores (demographic differences), and testing for the effects of role (patient or comparison), age, and role-by-age interactions.

**Results:**

The study sample of DMD (n = 285) and comparison (n = 292) participants provided open-text data: 577 wishes statements, 283 QOL-definition statements, and 149 goals statements. Inter-rater reliability (kappa = 0.77) reflected good agreement between different raters’ codes. Results suggested that people with DMD have aspirations that differ from their peers in several important ways. Both open-text and closed-ended data in both unadjusted and adjusted analyses generally showed that people with DMD were more focused on intrinsic aspirations such as health, healthcare, and independence than their peers. Compared to non-DMD persons, DMD individuals were much less focused on financial or housing concerns, community contributions, or spiritual growth. With age, patients’ aspirations focused less on extrinsic aspirations such as careers and work and increasingly emphasized emotion-oriented goals. Patients were markedly less likely to give a direct answer to the open-ended goals question.

**Conclusion:**

There were important differences in aspirations between people with DMD and their peers. These findings may be helpful for developing psychosocial interventions.

**Supplementary Information:**

The online version contains supplementary material available at 10.1186/s41687-022-00500-8.

## Introduction

Duchenne Muscular Dystrophy (DMD) is an X-linked disorder caused by mutation in the dystrophin gene. A rare (1 in 5050 live births [1–3]) neuromuscular condition which nearly always occurs in males,^1^ DMD is characterized by progressive muscle deterioration resulting in ambulation loss, decreased upper limb ability, and impaired cardiorespiratory function. Motor delay, abnormal gait, and disability in lower limb function are the most prevalent symptoms initially, which generally present between the ages of three and five years. Ambulation steadily decreases in the early stages of disease and is followed by a rapid and non-linear functional decline whereby progression to wheel-chair dependence occurs on average by early adolescence [2, 3]. Serious life-threatening complications can develop including scoliosis, disease of the heart muscle, and respiratory difficulties. DMD participants face profound uncertainty regarding lifespan, with mortality in the third or fourth decade [4], although medical advances have led to longer life expectancies [5].

This inevitable and progressive loss of independence due to DMD likely has an impact on life aspirations. While other children are typically focusing on excelling in sports, music, and/or academics, younger DMD patients are more often coping with the loss of important areas of function and suffer from a higher prevalence of cognitive impairment and executive functioning problems [6]. While their adolescent peers are exploring love and sexuality, DMD patients are longing for what might be unattainable [7]. While their adult peers begin to launch careers, partner, and start families, DMD patients are coming to terms with the emotional consequences of such challenging goals for themselves and for loved ones [8]. In contrast to their healthy peers, as they age DMD patients become more dependent on family-member caregivers and face ongoing uncertainty about their life expectancy [9]. The constant process of adapting to DMD entails reconsidering norms about adulthood [10], such as what ‘being a man’ means to them [11]. Because people with DMD can spend many years coping with the threat of imminent mortality, their aspirations will likely be different from people without a known foreshortened lifespan.

There is currently little to no research on how people with DMD differ from the general population in terms of limits on life aspirations. The present study aimed to fill these gaps via a cross-sectional case–control study that investigates aspirations in DMD participants as compared to a stratified comparison group of nationally representative children/adults. We focused particularly on age-related differences in aspirations, using qualitative and quantitative data that were collected to address the research questions.

## Methods

### Sample and procedure

Children aged 8 and older and adults (aged 18 and over) able to complete an online questionnaire were eligible to participate. This study recruited DMD participants via their parents/caregivers, who participated in an earlier study of DMD caregivers (see [12] for details). Comparison-group participants were recruited via IPSOS-Insight, a market research company (www.ipsos.com). The comparison group sampling frame was constructed such that it was nationally representative of the 2020 general United States (US) population ages 8–45, and to be balanced in age, race/ethnicity, gender, and US region. and were selected to accurately represent the United States population in age, race/ethnicity, gender, and region.

A web-based survey was administered October through December 2020 utilizing Alchemer, a HIPAA-compliant, secure web-based survey platform (www.alchemer.com). Recruitment was stratified by age group: 8–12, 13–17, and >  = 18, reflecting the above-described DMD disability trajectory [5, 13–15]. Although DMD can progress at varying rates, these age strata reflected the common phases of DMD progression: transitional-to-nonambulatory phase (up to age 12) and non-ambulatory phase (age >  = 13), with increasing dependence and involvement of other systems into adulthood (age >  = 18). Participants received honoraria to compensate them for their time completing the survey. Those with motor, visual, and/or other problems that made it difficult for them to complete the web-based survey instrument enlisted the assistance of a household member to enter their answers. The protocol was reviewed and approved by the New England Independent Review Board (NEIRB #20,203,038), and all participants provided informed consent before beginning the survey.

### Measures

Aspirations were measured using qualitative (open-ended) and quantitative (closed-ended) questions. The open-ended questions included: (1) Three Wishes [16], in which participants were asked, “If you could make three wishes, any three wishes in the whole world, what would they be?”; (2) Goals: “What are the main things you want to accomplish?”; (3) Quality of Life (QOL) Definition: “In a sentence, what does the phrase "Quality of Life" mean to you at this time?” The latter two are part of the QOL Appraisal Profile_v1_ [17]_._ The Three Wishes question has been used in previous research in 6–12 year olds comparing people with DMD to a comparison group [16].

In addition to the open-ended questions, 29 closed-ended goal-delineation items from the QOL Appraisal Profile_v2_ [18] were included. These rating-scale items queried a broad range of life domains (e.g., living situation, work/school, social relationships, health-related, spiritual, etc.), and asked about specific goals in each life domain. For example, a work/school item asked about whether the individual was concerned about keeping up at work or school. Response options enabled the respondent to indicate how much each goal statement was like them (1 = “not at all like me” through 5 = “very much like me”). Participants were given the option of not responding (Not applicable/Decline/I don’t know), which was coded as missing (− 99). These items are often analyzed individually to describe the person’s context [19, 20]. [The interested reader can contact the corresponding author for the QOLAPv2 Goal-Delineation items.]

Participants of all ages answered the Three Wishes open-ended question, whereas only participants aged 18 and older who did not opt for the Alternate survey answered the open-ended goals, QOL-definition questions, and the closed-ended goal-delineation items. This decision was based on the fact that these questions had not been validated in people under age 18.

Demographic Characteristics included year of birth, gender, year of diagnosis, and whether anyone in the household was or had been infected with the novel coronavirus-2019 (all participants).^2^Adult participants were asked about race, ethnicity, education, marital status, weight, height, with whom the person lives, employment status, and financial strain (difficulty paying bills).

#### Accommodating age and disability

Because of the broad age range of study participants and because DMD impacts multiple functional domains, the study design tailored the measures collected by age and/or participant preference. Teen or adult DMD participants were offered the option to choose the simpler child form of the survey if they felt they had trouble reading or concentrating. This “Alternate” survey contained fewer questions than the “Adult” survey. Additional file 1: Table S1 shows the questions administered by survey type.

### Qualitative and statistical analysis

#### Coding open-text data

The open-ended data were coded into goal themes by six trained raters (EB, RBB, AD, JBL, EK, MCF), according to an existing framework and then iteratively refined based on emergent themes in the open-text data described above. This existing framework provided a standardized protocol and comprehensive codebook originally derived using both deductive and inductive approaches in an extensive sorting procedure [21]. Themes in the current data were coded as “1” or “0” depending on whether they were reflected in the individual’s written text. As the goal-delineation themes were originally developed with a Human Immunodeficiency Virus (HIV) sample [21], which generally has different sociodemographic characteristics than the current study sample, some themes were not as prevalent here. Themes were added as needed for the current study, resulting in a set of 40 themes used for both the wishes and goals prompts and 17 for the QOL definition prompt. For each prompt, a theme of “No Direct Answer” was used if the respondent did not provide an answer or answered a different question than the one that was asked. This is distinct from leaving the question blank (i.e., skipping the question). For example, in response to the question “What are the main things you want to accomplish?” exemplary No-Direct-Answer responses were “seems rather great” or “nothing idk lol.”

Each text entry could be coded for as many themes as were reflected in the set for the corresponding prompt. Therefore, one entry could elicit one theme or more than one depending on its wording. For example, if one individual had written for their goal “My bills paid, my family healthy and happy, and family go to church,” it would have been coded as reflecting family welfare, financial concerns, health issues, mental health/mood state, and religious/spiritual concerns. In contrast, another individual’s goal was “Move to a different state,” which was coded with the single theme of living situation. Thus, we are assuming that the relevant factor here is the themes, not the individual wishes, goals, or QOL definitions themselves.

Training took place in two multi-hour sessions to understand the protocol and to utilize fully the codebook, where the themes were described fully and exemplified. Raters coded an initial set of ten participants’ data (from all three prompts), followed by a discussion of differences across raters. Incorporating exchanged feedback, they then coded the next ten participants’ data (again all prompts), and comparison and discussion now revealed almost no differences across raters. Raters coded data from 40 more responses (all three prompts), from which inter-rater reliability per prompt was computed in two ways on the 240 test responses (6 raters * 40 participant entries).

#### Inter-rater reliability

Two methods were used to assess aspects of inter-rater reliability. The first, Fleiss’s kappa [22] assessed degree of agreement over and above what would be expected by chance. This variant on the more familiar Cohen’s kappa [23] is used in cases of more than two raters. While there are no generally accepted rules of thumb for a desirable level of either form of kappa, some healthcare researchers have proposed values from 0.41–0.60 as “moderate,” 0.61–0.80 as “good,” and 0.81–1.00 as “very good.”[24, 25]

The second method assessed what proportion of the variance could be explained by the Rater effect. A low number is preferable as it reflects that the scores relate to the individual’s data being coded rather than reflecting a response style of the rater. This method used logistic regression to assess level of agreement among raters, with each of 240 “0” or “1” values regressed on the Rater variable, with its six rater-categories. High inter-rater reliability (IRR) for any given theme would be indicated by a nonsignificant Rater effect, and one that explained a low fraction of the variance in ratings (i.e., a pseudo-R-squared in the low single digits).

#### Comparing length, number of themes, and inter-method associations

Analysis of Variance (ANOVA) models were used to compare length of open-text response and number of themes (dependent variables) by role (patient or comparison; independent variable) to compare the complexity of the responses by group.

#### Differences in age distributions by role

The child, teen, and adult data sets revealed age differences between patients and comparisons: there were differences in mean age, the frequency of certain age ranges, and the shape of the age distributions. We decided that, in addition to adjusting for age, in our models we would apply weights so as to simulate more comparable age distributions. We developed a weighting variable that reduced the disproportionate impact of comparison group participants of specific ages so that the age distributions between patients and comparisons would be much more similar. For example, 24–34-year olds were far over-represented amongst comparisons; these were thus down-weighted (i.e., treated as 0.4 of a participant instead of 1.0 participant), not excluded, for multivariate analysis. While the weighting might not completely eliminate the age differences between the two groups, it was conducted to make those distributions comparable enough to render the planned analyses tractable [26].

### Analyzing the aspirations data

#### Unadjusted comparisons

Descriptive statistics summarized either the proportion of each group coded as reflecting a given theme for the open-text data or the mean and standard deviation for the closed-ended goal-delineation items. Effect size was summarized by phi for comparison of proportions, or Cohen’s d for comparison of means, for the open- and close-ended data, respectively.

#### Adjusted comparisons

Propensity scores were used to control for demographic differences other than age, between DMD participants and comparison participants in the below-described multivariate models [27]. The goal of the propensity-score modeling was to create a score for covariate adjustment across all age groups, thereby allowing us to compare aspirations across the age span. This was the central contribution of the present work. We thus used the following pragmatic approach for dealing with the fact that some covariates were simply not asked and thus not available (see Additional file 1: Table S1). Accordingly, our propensity-score model adjusted for those covariates that differed between patient and comparison groups in bivariate analyses described below. Separately for adults and for teens/children, a logistic regression model was computed predicting the dependent variable Role (DMD patient vs. comparison participant) from the applicable covariates. For adults who completed the adult survey, the covariates included the following: ethnicity, White race, Black race, region, marital status, difficulty paying bills, whether currently working, education, whether received help completing survey, and whether someone in household had contracted COVID-19. Only male comparison participants were included, since all DMD participants were male. For children, teens, and those who completed the Alternate survey, the covariates included the following: ethnicity, White race, Black race, region, whether received help completing survey, and whether someone in household had contracted COVID-19. For a small proportion of participants (2%), propensity scores were based on the mean propensity score among the individual’s age group.

Multivariate models were then computed to hone the contrasts, comparing the patient and comparison groups on binary themes for the coded open-text data (wishes, QOL definition, goals), or on the rating-scale close-ended goal-delineation items from the QOLAP_v2_. Because of our particular focus on age-related differences in aspirations, the models evaluated how patients vs. comparison participants (i.e., ‘role’) differed in aspiration outcomes after adjusting for their propensity scores, age, and the role-by-age interaction. Logistic regression was used to analyze the individual themes for the coded open-ended data, while Analysis of Covariance (ANCOVA) was used to analyze the closed-ended goal-delineation data.

For logistic regressions, 13 of the 95 theme variables showed no variation and thus were excluded from analyses. Further, some of those analyzed showed complete or quasi-complete separation in logistic regression, and for these we reported only descriptive results.

#### Interpretation of main effects in the context of interactions

The abovementioned multivariate models aimed to investigate how patients differed from comparisons in their Aspirations and at different ages, after adjusting for demographic variables that might have confounded the raw descriptive comparisons. Interpreting main effects can be challenging when the model contains interaction terms, because the latter are collinear with the former. To address this challenge, plots of substantial interaction effects were used to facilitate their interpretation. In order to display an interaction effect (Role*Age), we created scatterplots that graphed predicted values from the model (Y-axis) against age (X axis). Any theme variable with a group mean that was < 0.01, or with a |Beta| or |Estimated Beta| out of the usual range (> 1.3), was excluded from interaction graphs.

#### Interpretation in the context of many contrasts

The present study involves a large number of statistical contrasts primarily because it is investigating research questions that have not been addressed to date and which involve translating nuanced qualitative data into quantitative metrics. In demographic comparisons, we relied on p-values to identify group differences, but in other analyses we focused our interpretation on effect sizes (ES). Cohen’s criteria were used to facilitate interpretation of differences for medium and large effect sizes, respectively: in proportions (Phi of 0.3 and 0.5); in mean differences (Cohen’s *d* of 0.5 and 0.8); in model explained variance (R^2^ of 0.06 and 0.14); and in model parameter estimates (standardized beta coefficients of 0.3 and 0.5). While we report ES regardless of magnitude, we interpret only medium or large ES because these are generally considered clinically important [28]. Tables are conditionally formatted using data bars in unadjusted comparisons, and using different colors and saturation levels in adjusted comparisons to highlight effects’ direction and magnitude.

IBM SPSS version 27 [29] and the R software [30] were used for all analyses.

## Results

### Sample

The study sample included 285 people with DMD and 292 comparison participants. Table 1 shows descriptive information about the two subsamples, as well as results of analyses contrasting patients vs. comparison on all demographic variables. The majority of the sample was Caucasian, 8–18% were Black, and 10–20% were Hispanic. Both subsamples had a normal body mass index on average. Study participants in both groups were young adults from a variety of regions of the contiguous United States. Mean ages for patients vs. comparison were, unweighted, 17.3 and 18.1 years, respectively, and weighted, 17.3 for both.

**Table 1:** Sample demographic characteristics (N = 577)

	Patient (N < = 285)		Comparison (N < = 292) ¥		Cohen's *d*	Cramer's V	p
Variable	Mean	SD	Mean	SD			
Age	17.3	5.4	18.1	7.5	0.1		0.150
Body mass index	24.0	6.0	22.8	5.5	− 0.2		0.020
Years since DMD diagnosis	13.6	5.2	na	na			

Variable	#	%	#	%			
Male	285	100%	292	100%			
Living alone*							
Yes	0	0%	1	0%		0.05	0.330
Marital status (from adult survey)**						0.39	< .0005
Never married	87	99%	53	71%			
Married	1	1%	14	19%			
Cohabitation	0	0%	7	9%			
Separated	0	0%	0	0%			
Divorced	0	0%	1	1%			
Widowed	0	0%	0	0%			
Ethnicity*							
Hispanic or Latino	26	10%	58	20%		0.15	0.001
Race (check all that apply)							
White	246	86%	220	75%		0.14	0.001
Black or African American	22	8%	52	18%		0.15	< 0.0005
Other	9	3%	32	11%		0.15	< 0.0005
Missing	8	3%	0	0%			
United States Region						0.17	0.048
East North Central	28	10%	49	17%			
East South Central	19	7%	18	6%			
Middle Atlantic	33	12%	38	13%			
Mountain	14	5%	15	5%			
New England	7	2%	13	4%			
Pacific	61	21%	33	11%			
South Atlantic	66	23%	61	21%			
West north central	14	5%	17	6%			
West south central	27	9%	29	10%			
Non-contiguous	0	0%	0	0%			
Missing	16	6%	19	7%			
Difficulty paying bills (from adult survey)***						0.34	0.004
Not at all difficult	57	72%	25	46%			
Slightly difficult	13	16%	8	15%			
Somewhat difficult	6	8%	12	22%			
Very difficult	3	4%	7	13%			
Extremely difficult	0	0%	2	4%			
Employment status (from adult survey)						0.74	< 0.0005
Employed	4	5%	36	68%			
Unemployed	56	66%	14	26%			
Retired	0	0%	3	6%			
Disabled due to medical condition	25	29%	0	0%			
Education (from adult survey)						0.35	0.005
Less than 12th grade	2	2%	3	6%			
High school diploma	31	37%	16	30%			
Some college	33	39%	14	26%			
Technical (Vocational) degree	10	12%	2	4%			
4-year University degree	7	8%	12	23%			
Masters degree	1	1%	6	11%			
Doctoral or Professional degree	0	0%	0	0%			
Had help completing survey*							
Yes	141	49%	69	24%		0.27	< 0.0005
Participant or a household member had COVID-19						0.30	< 0.0005
Definitely or probably yes	3	1%	56	19%			
No	277	97%	231	79%			
Missing	5	2%	5	2%			

Some sets of percentages may not add up to 100% due to rounding.GED = General Educational Development (i.e., high-school equivalency test) SD standard deviation

*For these variables a non-response was counted as the absence of the characteristic in question

**For this variable a non-response was treated as Never married

***Cramer's V and p are based on non-missing results

¥ Comparison participants included only if age < 35

DMD participants were all male, diagnosed on average 13 years before, and, among adults, had a median level of education of “some college.” Sixty-six percent of participants over age 18 were unemployed, 29% were unable to work due to their medical condition, and 5% were employed. About half received help completing the survey (Table 1). The adult DMD participants endorsed little to no difficulty paying bills (88%), almost all were unmarried, and none lived alone. Only 1% reported that a household member had contracted COVID-19.

The comparison subsample also had a median level of education of “some college,” but a higher proportion reported completing a 4-year college degree or graduate/professional degree (Table 1). Sixty-eight percent of adults were employed, 26% unemployed, and there were no reports of being unable to work due to a medical condition. Sixty-one percent endorsed little or no difficulty paying bills. Almost three-quarters reported being unmarried, and only one person lived alone. Nineteen percent reported that a household member had had COVID-19. About one-fourth received help completing the survey.

Analyses contrasting these two groups on the demographic variables revealed significant differences (*p* < 0.05) on body mass index, marital status, ethnicity, race, region, difficulty paying bills, employment status, education, whether a household member had contracted COVID-19, and whether they received help with the survey (Table 1). DMD participants reported less financial strain and a substantially lower incidence of COVID-19 in the household (Table 1). DMD participants were more likely to receive help completing the survey.

### Qualitative coding reliability

The open-text data included 577 wishes statements, 283 QOL-definition statements, and 149 goals statements. Inter-rater reliability was computed for a randomly selected subset of 17 topics of the 93 across these three prompts (see Additional file 1: Table S3).^3^ The mean kappa across the topics was 0.77 (SD = 0.17, range 0.51 to 0.98), reflecting a good level of agreement [24, 25]. The best estimates for pseudo-R^2^ for rater, averaged over the Cox & Snell and Nagelkerke methods, had a mean of 0.021 (range 0.001 to 0.104), with 0.17 < *p* < 1.00, suggesting that any rater effect on the coded themes was negligible.

### Propensity scores

Additional file 1: Table S2 shows model descriptive statistics as well as parameter estimates for all covariates, from the propensity-score model distinguishing patients from comparisons. Our propensity-score model adjusted for those covariates that differed between patient and comparison groups (see Table 1). Averaged across iterations, the model explained 49% of the variance as estimated via pseudo-R^2^.

### Aspiration differences by role: bivariate results

#### Open-text data

Table 2 provides the descriptive statistics on the themes coded in the three open-ended aspirations prompts, and Table 3 provides them for the closed-ended goal items by role. Depending on the prompt, 0 to 43% of DMD participants and 0 to 20% of comparison participants had missing responses in the open-ended questions. Four themes showed medium or large effect-size differences in proportion by role (|Phi|≥ 0.3). Unadjusted estimates suggest that patients were much more likely than comparison participants to mention wishes related to health issues, and much less likely to mention financial concerns. Similarly, DMD participants were much less likely than comparison participants to mention financial goals.

**Table 2:**
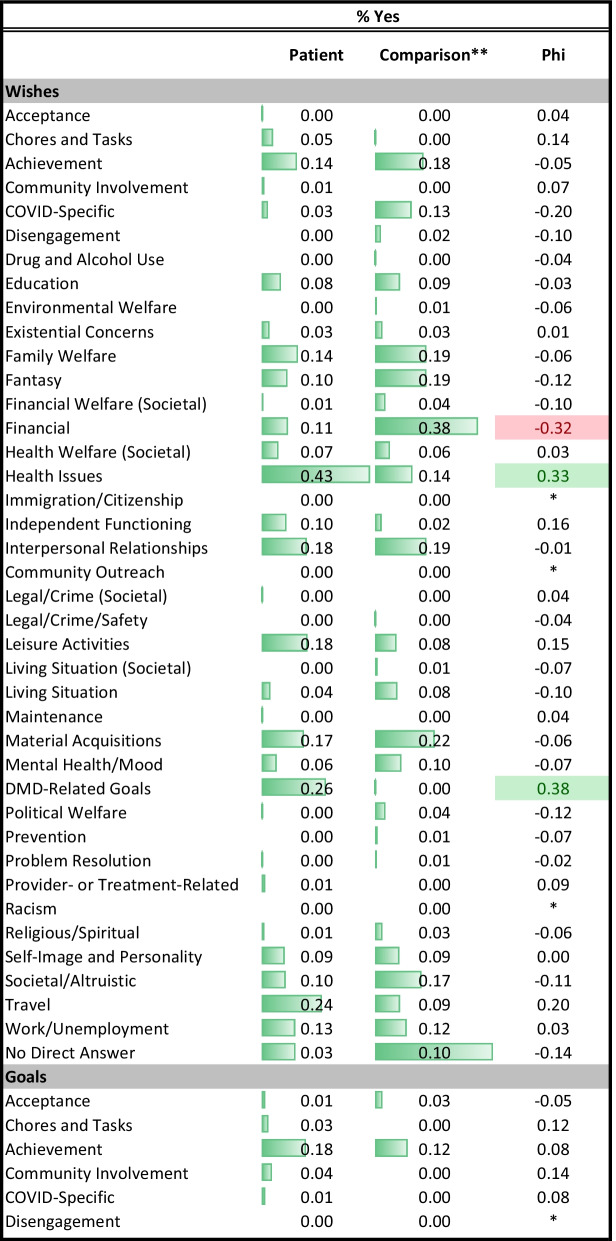

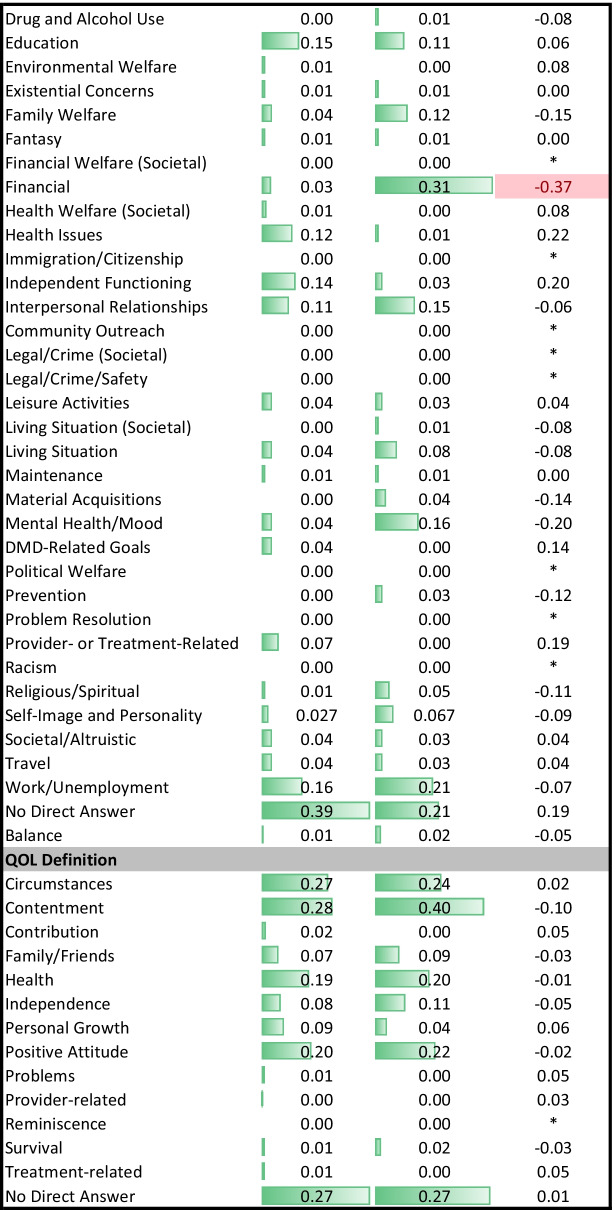
Descriptive statistics for themes from open-text prompts

**Table 3:**
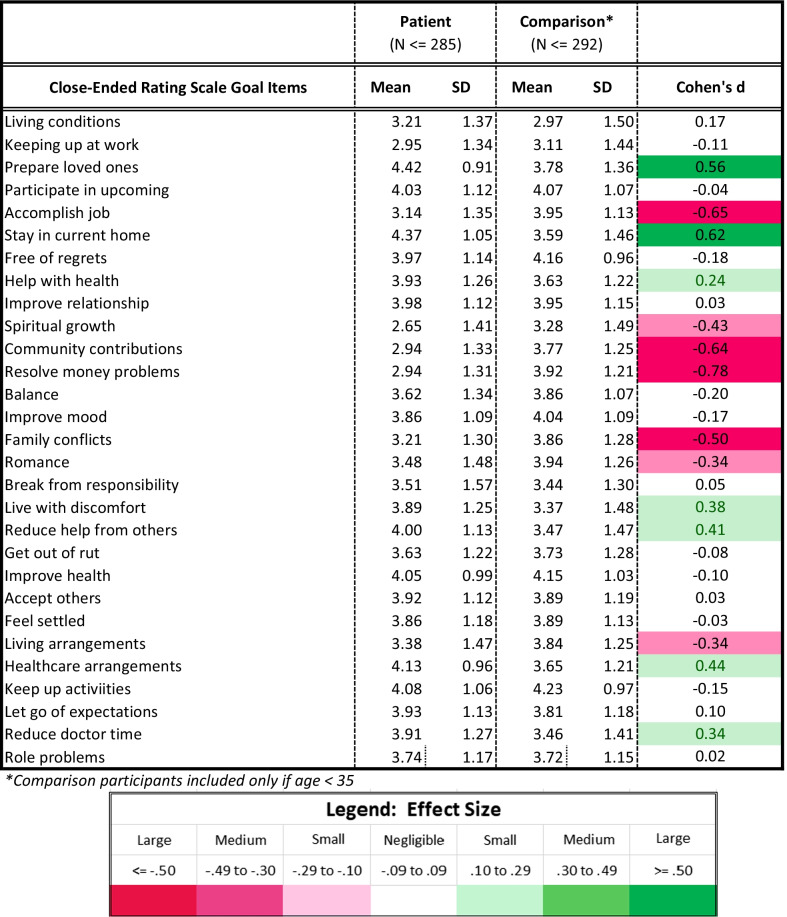
Descriptive statistics for close-ended goal items

#### Close-ended goal items

**Fig. 1:**
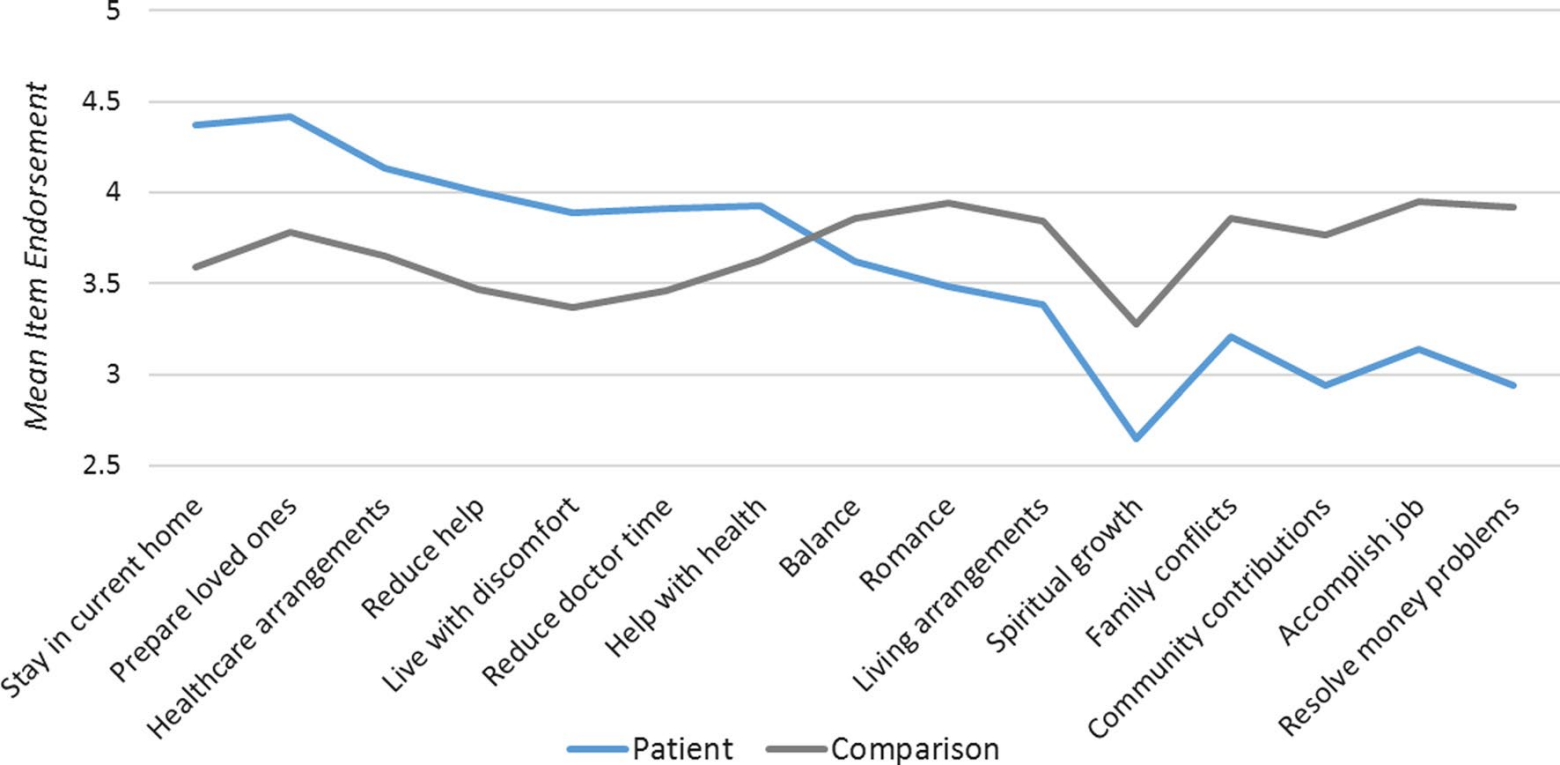
Goal item endorsement by role. Unadjusted means are shown for patients (blue line) and comparison participants (grey line) on the close-ended goal items with the most substantial differences

Figure 1 shows unadjusted mean differences on the close-ended goal items that showed the most substantial differences (|Cohen’s d|≥ 0.20) between adult DMD participant and comparison groups. Where mean endorsement was higher for DMD participants, the highest between-group effect sizes were for staying in their current home (ES 0.62), preparing their loved ones for a time when their health would be worse (ES 0.56), and maintaining healthcare arrangements (ES 0.44) (Table 3). Where mean endorsement was lower for DMD participants, highest between-group effect sizes were accomplishing a job (ES − 0.065), community contributions (ES − 0.64), resolving money problems (− 0.78) and family conflicts (ES − 0.50).

### Aspiration Differences by Role: Multivariate Results

#### Open-text data

Table 4 shows results of the multivariate models comparing patients and comparison participants on coded themes from their open-text Aspirations data, after adjusting for propensity scores, age, and role-by-age interactions. Models with R2 ≥ 0.06 (medium ES) are described. To facilitate interpretations of interaction effects, for all models with medium or large interaction ES, plots depict predicted values.

**Table 4:**
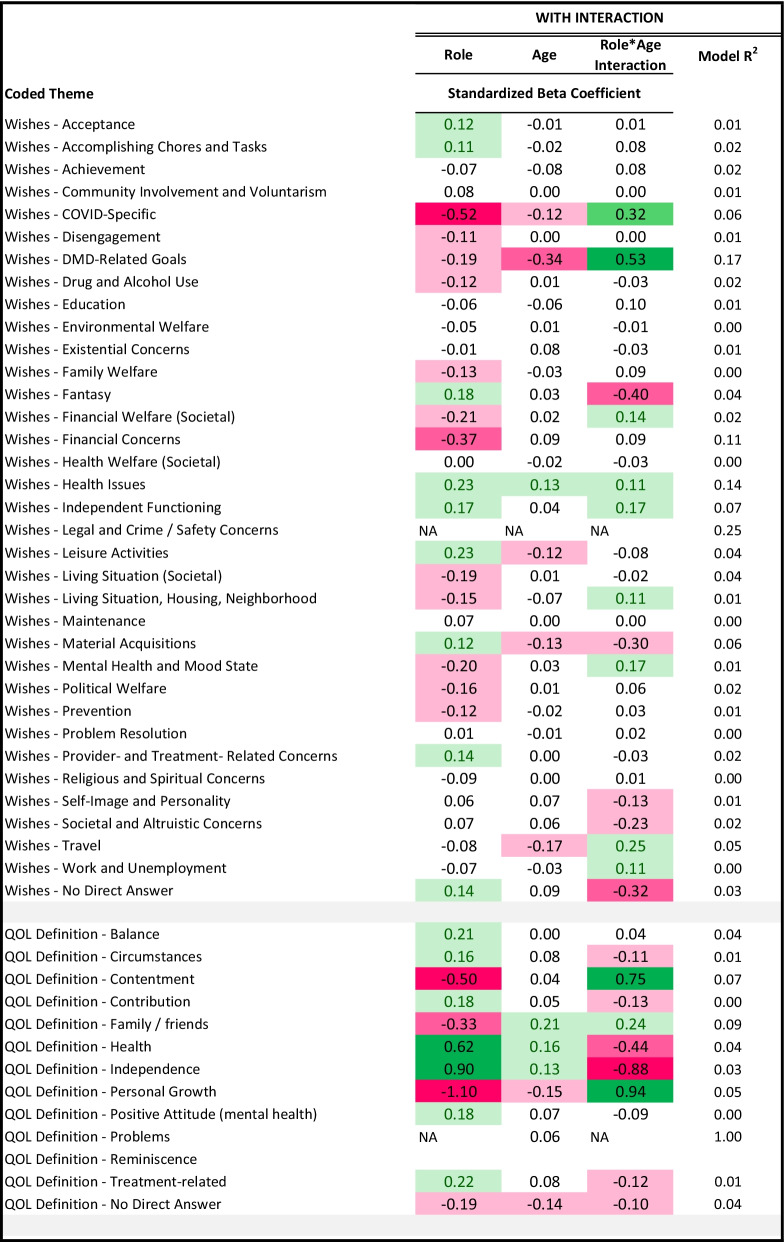

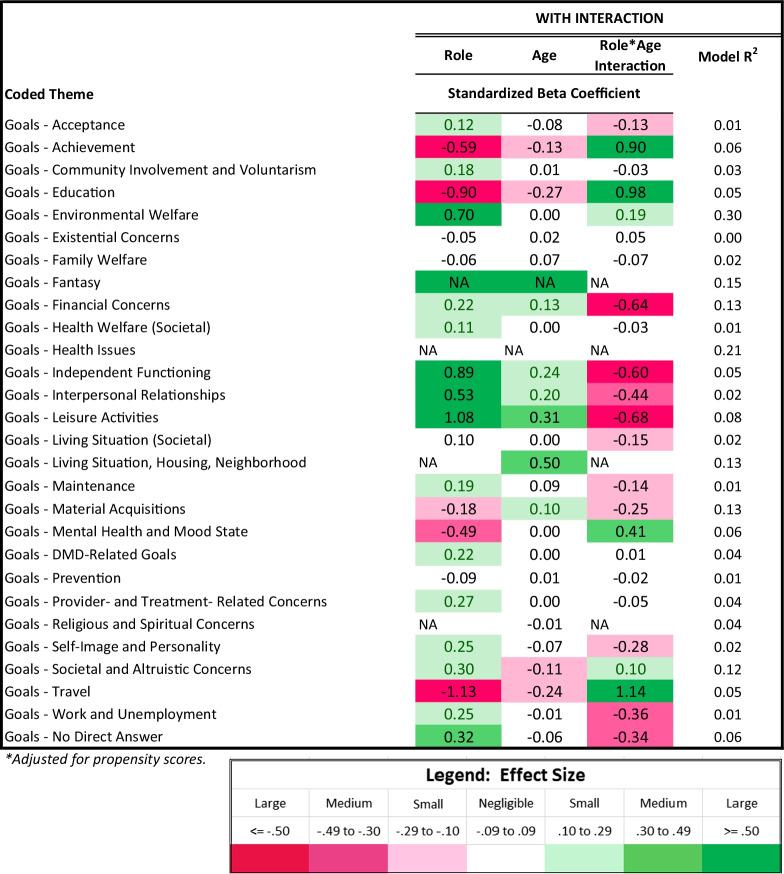
Results of patient vs. comparison groups' multivariate logistic models predicting coded themes*

**Fig. 2:**
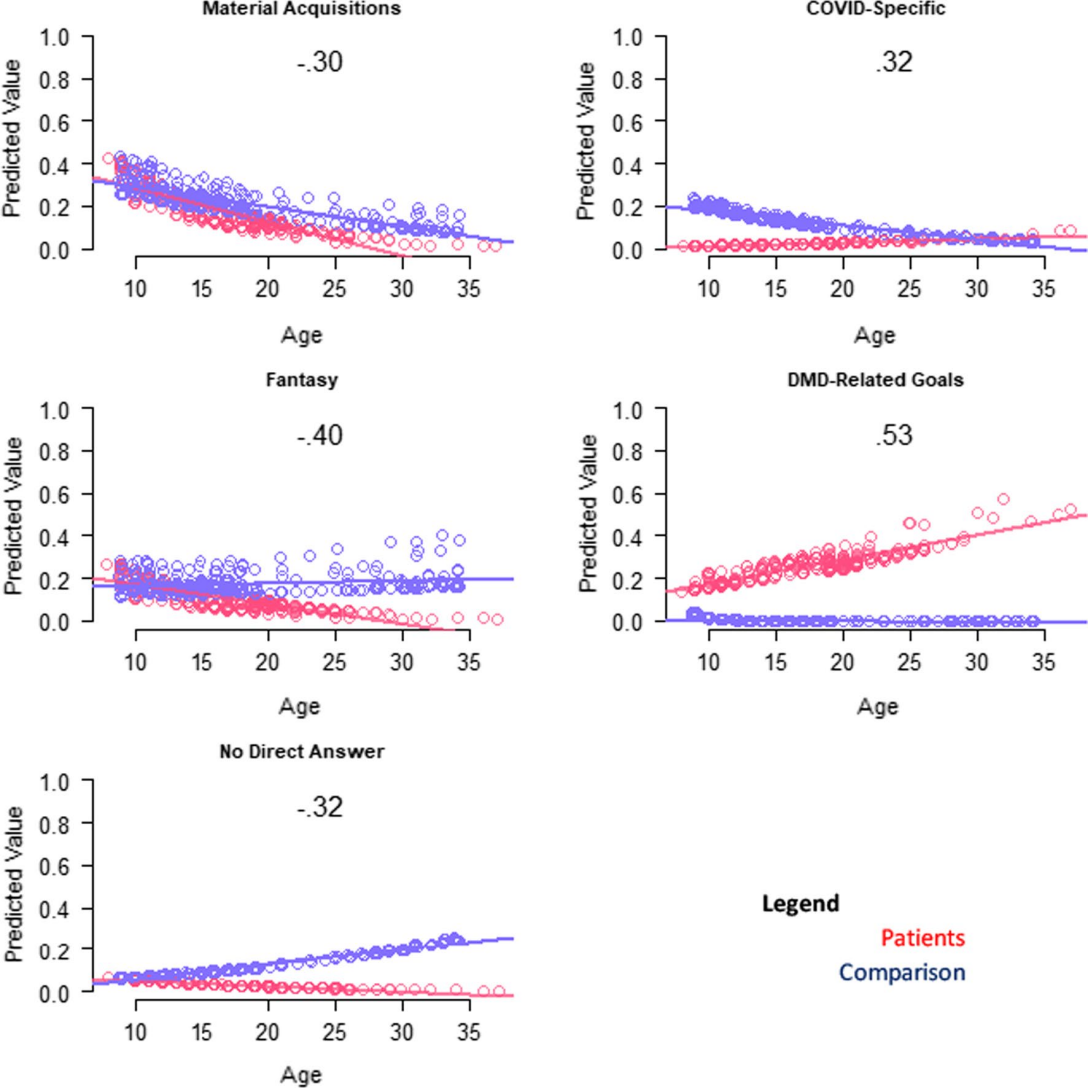
Interaction plots for adjusted logistic models predicting wishes themes. Role-group differences for medium- and large-ES interaction effects are shown. Patient predicted values are shown in red; comparison in blue

The “wishes” models that explained the most variance involved DMD-related goals, health issues, financial concerns, independent functioning, material acquisitions, and COVID-specific concerns (R^2^ 0.17 to 0.06; Table 4). DMD participants were more likely to mention wishes reflecting DMD-related concerns, particularly at higher ages (Fig. 2). DMD participants were less likely to mention COVID-specific concerns, material acquisitions, or content reflecting fantasy; or to give no direct answer, particularly at older ages (Fig. 2). Further, comparison participants were more likely to mention financial concerns.

**Fig. 3:**
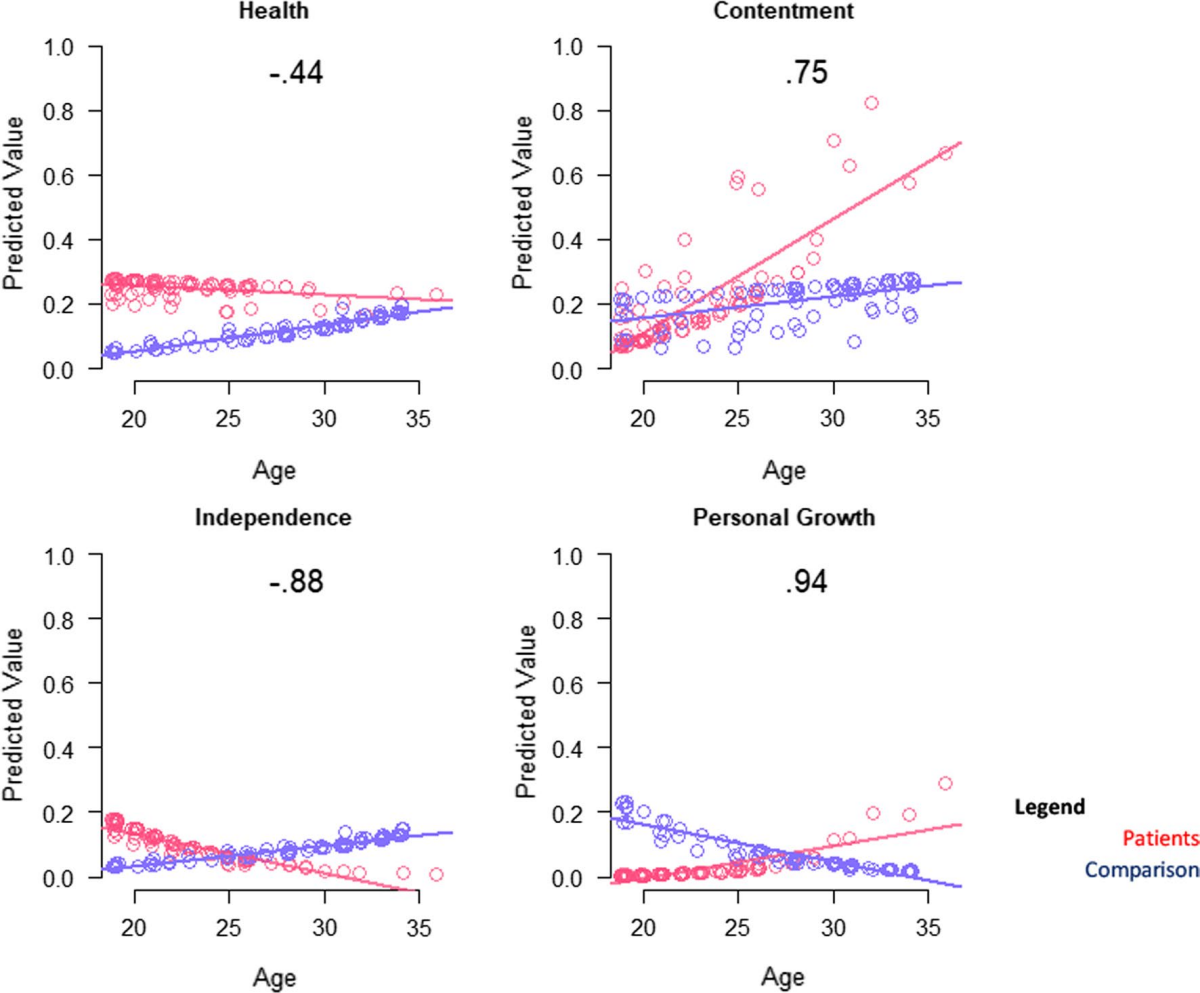
Interaction plots for adjusted logistic models predicting QOL definition themes. Role-group differences for medium- and large-ES interaction effects are shown. Patient predicted values are shown in red; comparison in blue

The QOL definition models that explained the most variance involved family/friends and contentment (R^2^ 0.09 to 0.07; Table 4). Patients were more likely to mention a QOL definition reflecting contentment or personal growth, particularly at older ages (Fig. 3). Although health and independence were more prominent in younger adults with DMD, older participants were less likely to mention such themes when thinking about QOL definition (Fig. 3).

**Fig. 4:**
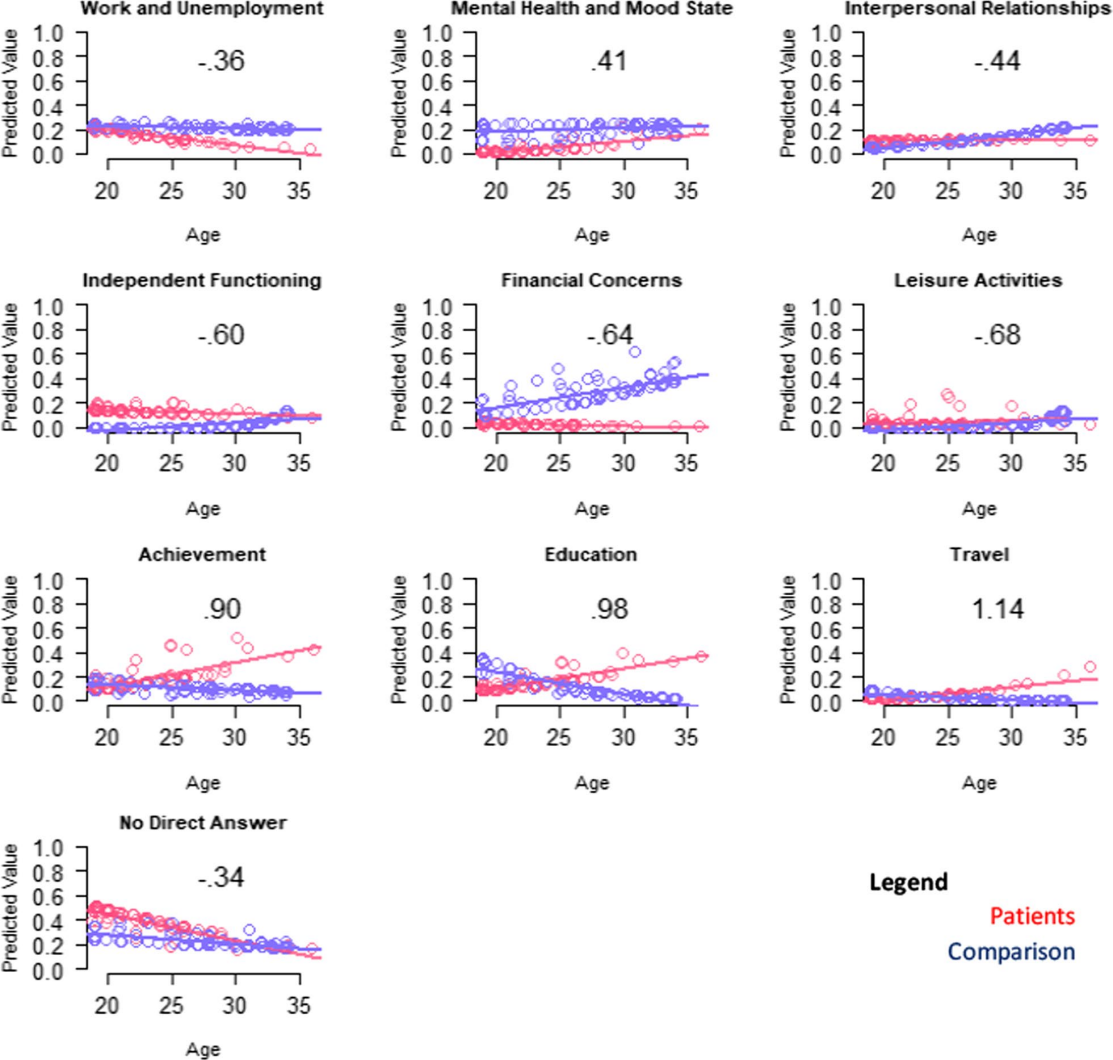
Interaction plots for adjusted logistic models predicting Goal themes. Role-group differences for medium- and large-ES interaction effects are shown. Patient predicted values are shown in red; comparison in blue

The “goals” theme models that explained the most variance involved environmental welfare, financial concerns, societal/altruistic, mental health/mood, and no direct answer (R^2^ 0.30 to 0.06; Table 4). DMD participants were more likely, particularly at higher ages, to mention goals related to travel, education, and achievement (Fig. 4). They were less likely, particularly at higher ages, to mention goals related to work/unemployment or to give no direct answer (Fig. 4). While patients were overall more likely than comparison participants to mention independent functioning, interpersonal relationships, and leisure activities, these concerns were similarly pertinent to older patients but became more important for older comparison participants. While patients were overall less likely than their peers to mention mental health/mood state, this difference narrowed for older participants (Fig. 4). Patients were, however, more likely overall to mention goals related to environmental welfare and societal/altruistic concerns. Financial concerns were always less prominent for DMD participants and became more important for comparison participants with age.

#### Close-ended goal items

**Fig. 5:**
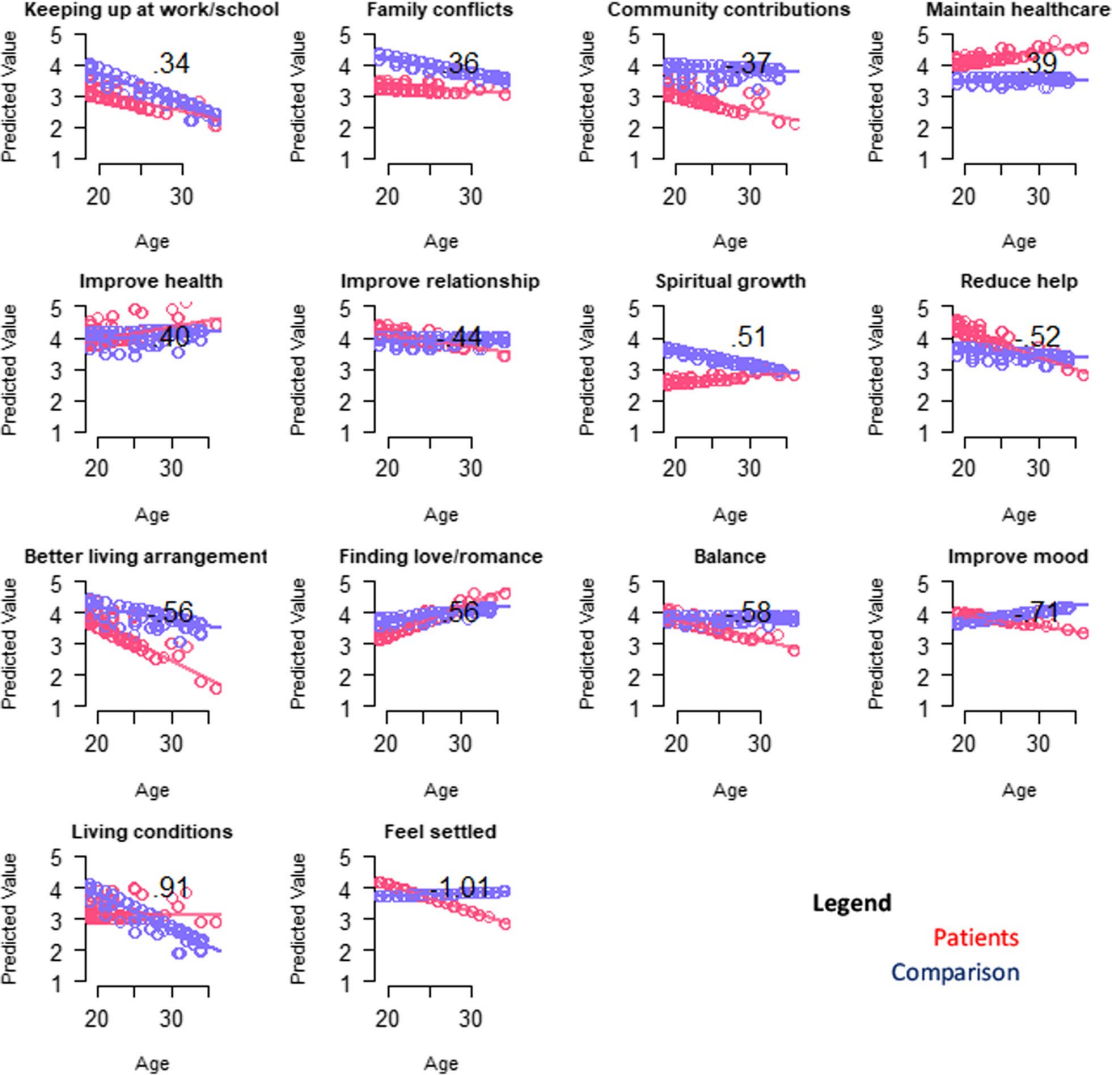
Interaction plots for adjusted ANCOVA models predicting Goal items. Role-group differences for medium- and large-ES interaction effects are shown. Patient predicted values are shown in red; comparison in blue

Table 5 and Fig. 5 show results of the multivariate models comparing patients and comparison participants on close-ended goal items, after adjusting for propensity scores, age, and role-by-age interactions. Patients, particularly at higher ages, were more likely to endorse goals related to maintaining healthcare. There were several topics which DMD participants or comparisons were more likely to endorse, and which became less or more important at higher ages. Goals which became more important with age for DMD participants were related to participating in upcoming important events, accepting others, reducing time with doctors, and getting out of a rut. Older comparison participants emphasized problems with living conditions, finding better living arrangements, resolving family conflicts, seeking spiritual growth, and keeping up at work/school. Goals which became less important with age for DMD participants were related to reducing help from others, balance between obligations and enjoyable activities, improving mood, feeling settled, improving relationships, and community contribution. Finding love/romance and improving health were less important for young-adult DMD participants and became more important at higher ages and more similar to non-DMD participants.

**Table 5:**
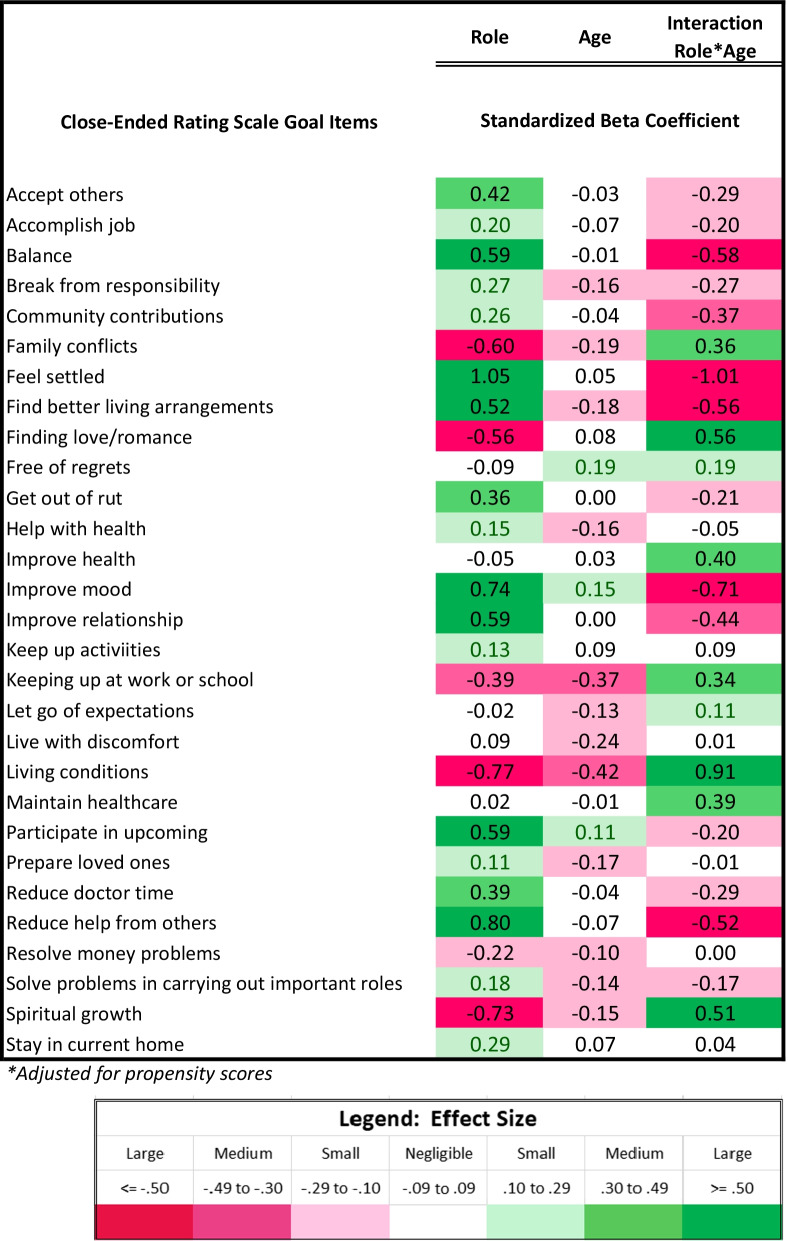
Results of patient vs. comparison groups' ANCOVA models predicting close-ended goal items*

## Discussion

Our study suggests that people with DMD have aspirations that differ from their peers in several important ways. Both open-text and closed-ended data, in both unadjusted and adjusted analyses, generally suggested that people with DMD were more focused on health, healthcare, and independence than their peers. They were much less focused on financial or housing concerns, community contributions, or spiritual growth.

Our study also showed that academic and achievement-related aspirations were cited by DMD participants less frequently than by the comparison group—up until their early twenties, after which these aspirations took on greater prominence. In contrast, aspirations specifically related to employment were less frequently endorsed by DMD participants at higher ages. Thus, unlike in the general population, for people with DMD, academic- and achievement-related aspirations appear to be distinct from employment. A substantially lower proportion of DMD participants reported being employed (26% versus 66%), with approximately a third of the DMD participants noting their disability as impacting the ability to work. This is consistent with prior research showing that chronic illness disrupts the course of work trajectories, particularly with young age at diagnosis and longer illness duration strongly impacting the chances of participating in the labor market [31].

Despite the increased importance of education, DMD participants were less likely than comparison participants to have completed a four-year college degree or master’s program (8% versus 23%, and 1% versus 11% respectively). A report by Hamdani et al. mentions the normative assumptions regarding adult roles in patients living with DMD, particularly academic achievement and work [5]. Pursuing such aspirations is important for establishing adult identities, despite the limits on DMD participants’ independence and work experience.

Self-determination theory has researched the relative strength of intrinsic aspirations (e.g. relationships, personal well-being, community) versus extrinsic aspirations(e.g. financial wealth, fame, image) [32, 33]. Self-determination theory has highlighted the negative psychological consequences of placing a disproportionate emphasis on extrinsic versus intrinsic aspirations [34]. Our findings suggest that DMD results in a greater focus on short-term, emotion-oriented aspirations, conducive to closeness and activities with family and friends. For example, with age, patients’ aspirations appear to center less on work and more on hedonic concerns such as contentment, personal growth, and accepting others.

Curiously, DMD participants were substantially less likely than the comparison group to supply a direct answer to the open-ended aspirations question (61% vs 86%, respectively). There may be a number of reasons underlying this relatively low proportion of DMD patients answering the open-ended goal questions. For example, they could have had more difficulty responding to open ended questions as they are cognitively more complex, require typing, and, for patients whose caregivers are completing, they might be less apt to share extensively given caregiver is present. It may, however, reflect an important aspect of the experience of living with DMD: that one does not think as much about what one wants to accomplish long-term. Having aspirations, even if short-term, is important for one’s sense of purpose and meaning in life [35, 36], and thus can be effective in preventing depression [37, 38]. This subtle finding is thus worthy of proactive and preventative intervention by rehabilitation professionals involved in the care of people with DMD.

Our findings also have implications for measurement of constructs like QOL. Many factors relevant to DMD are inadequately assessed by commonly used, generic, preference-based QOL instruments [39] or validated measures of work productivity [40]. These measures do not fully capture the impact of the disease. They miss a host of relevant constructs, including but not limited to emotional effects of disease, participation in society, access to care, aspirations, expectations of future relationships, worry about the future and family [41], and issues particularly salient at the transitional stage of the disease. Our findings on differences in aspirations in people with DMD versus comparison participants would lend support to a more comprehensive measurement strategy in DMD research and perhaps in research on other chronic diseases.

The present study has many advantages. It is, to our knowledge, the first study of aspirations of people with DMD that includes an age-stratified comparison cohort of non-DMD peers. The study measures triangulate on the notion of aspirations, with open- and closed-ended prompts querying related concepts, and documents these differences across the lifespan from middle childhood to middle age. A further advantage of the present study is its large sample size, which seems aptly representative of the United States population, thereby facilitating the generalizability of its findings. It also employs rigorous implementation of mixed methods, juxtaposing unadjusted and adjusted models, which help tease apart what differences have competing explanations in demographic and developmental factors. We found, for example, many substantial role-by-age interactions which revealed that group differences in aspirations differ according to age. Such complexities are important to consider when applying this study’s findings to providing support to patients and their families.

Despite the above strengths, the limitations of the present study must be acknowledged. First, the study design allowed patients to opt out of the more comprehensive survey due to self-perceived limitations in being able to read or pay attention. This design element was deemed ethically important because of the elevated prevalence of cognitive problems and attention-deficit concerns in people with DMD [14]. As a result, although all ages responded to the wishes prompt, among DMD participants, only adults who did not in effect self-evaluate as cognitively challenged provided data on the other open-ended and closed-ended prompts.

Second, while we believe that adjusting for demographic factors has helped us to isolate group differences, there is some debate as to how well sizable pre-existing group differences can effectively be neutralized using multivariate control [26]. The demographic comparisons revealed that patients and their peers differed nontrivially on marital status, difficulty paying bills, employment status, and education level. The study attempted to adequately address these differences through the judicious use of propensity scores. Nonetheless, the use of propensity scores in case–control studies may suffer from artifactual effect modification of the odds ratio by level of the propensity score and may not fully adjust for measured confounding factors [42]. Additionally, the missing-data imputation method used may underestimate the variance of the parameter estimates and thus inflate Type I errors [43]. Covariate adjustment using propensity scores assumes that the nature of the relationship between the propensity score and the outcome has been correctly modeled ([27], p. 409). The plots illustrating our findings show that the observed data points are close to the regression lines, thereby supporting the idea that the outcome has been correctly modeled and assuaging concerns about bias generated by the pragmatic approach used to generate propensity scores across the full age range of the study sample. If we had implemented models separately for those with and without adult-level covariates (e.g., marital status, difficulty paying bills, etc.), we would likely have missed important interaction effects. Although a mean imputation approach is not standard for propensity scores, there was only small amount of missing data. Thus, our pragmatic approach for handling this complex dataset structure enabled filling a gap in the literature. Finally, due to the number of variables generated from the content analysis of qualitative data, a large number of comparisons were done. We have attempted to mitigate the possible multiple-comparison issue by focusing on only medium and large ES using Cohen’s criteria [44]. Given the sample sizes of the two groups being compared, the study has sufficient statistical power to detect even a small ES with an alpha level of 0.05 [44]. Thus, medium and large ESs would have an even smaller Type I error rate. As this study presents for the first time to our knowledge, novel results about how DMD patients’ aspirations differ from a comparison sample, we believe that quantitatively considering all the data gleaned from the qualitative analysis is worthwhile and important.

Third, due to differences in the elicitation process of the open- and closed-ended prompts, the results may not always seem aligned. For example, patients seemed less likely to make statements reflecting interpersonal or family themes in the open-text prompts, but in the closed-ended statements they were more likely to endorse improving relationships and accepting others. This difference may reflect the distinction between recall and recognition in neuropsychology [45, 46], where the former elicits more prominent concerns (i.e., areas with difficulty) and the latter elicits a more standardized set of areas to be rated (i.e., all areas are displayed and rated). This complexity is an advantage of mixed-methods research, but results are less easily summarized in “sound-bites.” Finally, a higher proportion of DMD participants, had No Direct Answer responses in the open-ended goal question. The cause for this differentiation may have been due to the inability to identify goals, comprehension of the question, or influence from the help received to complete the survey. This finding warrants further evaluation.

### Conclusions

In summary, this is the first study to assess the differences in the reporting of goals and aspirations between individuals with and without DMD. The large sample size of participants facilitated the ability to examine how aspirations change as individuals age, which is of particular importance for progressive diseases such as DMD. When attempting to ascertain the humanistic impact of disease, this study highlights the value of incorporating its impact on aspirations using both qualitative and quantitative data, particularly for diseases with early onset. The methodology applied in this study can be leveraged for future assessments in DMD and other diseases. This study provides insight into important differences in aspirations between people with DMD and their peers. These differences encompass not just health and independence, but also more hedonic and intimacy-related concerns. This unique study points to important potential foci for physical-therapy interventions (i.e., independence), as well as areas for coaching interventions to improve QOL, such as helping patients to identify meaningful and viable goals and to affect desired changes in hedonic and intimacy-related concerns.

## Supplementary Information

**Additional file 1. Supplemental Table 1.** List of Measures by Age Cohort. **Supplemental Table 2**. Propensity Score Model. **Supplemental Table 3**. Inter-rater Reliability Results

## Data Availability

The study data are confidential and thus not able to be shared.

## Abbreviations

ANOVA: Analysis of variance
DMD: Duchenne Muscular Dystrophy
ES: Effect size
QOL: Quality of life
QOLAP_v2_: QOL Appraisal Profile version 2
US: United States

## Footnotes

1 Females can be carriers and may have mild symptoms, such as mild but progressive muscular weakness and increased serum creatin kinase levels [1]. The DMD phenotype in female carriers of a dystrophin mutation has a direct correlation with a skewed X-chromosome inactivation pattern [1].

2 As the data collection took place during the COVID pandemic, infection status would be a stressor that should be tracked in any study of QOL.

3 We deemed that this subset of about a fifth of the data was adequate to assess inter-rater reliability.

## Code availability

Requests for software code will be considered, and will be made available if deemed reasonable.

## References

[1] Giliberto F, Radic CP, Luce L, Ferreiro V, de Brasi C, Szijan I (2014). Symptomatic female carriers of Duchenne muscular dystrophy (DMD): Genetic and clinical characterization. J Neurol Sci.

[2] Jones HR, Darryl C, Darras BT (2003). Neuromuscular disorders of infancy, childhood, and adolescence: a clinician's approach.

[3] Services UDHAH (2015) Duchenne muscluar dystorphy and related dystrophinopathies: developing drugs for treatment. Guidance for industry. Draft. Silver Spring, MD U.S. Department of health and human services food and drug administration center for drug evaluation and research (CDER) Center for biologics evaluation and research (CBER)

[4] Szabo SM, Salhany RM, Deighton A, Harwood M, Mah J, Gooch KL (2021). The clinical course of Duchenne muscular dystrophy in the corticosteroid treatment era: a systematic literature review. Orphanet J Rare Dis.

[5] Hamdani Y, Mistry B, Gibson BE (2015). Transitioning to adulthood with a progressive condition: best practice assumptions and individual experiences of young men with Duchenne muscular dystrophy. Disabil Rehabil.

[6] Wingeier K, Giger E, Strozzi S, Kreis R, Joncourt F, Conrad B (2011). Neuropsychological impairments and the impact of dystrophin mutations on general cognitive functioning of patients with Duchenne muscular dystrophy. J Clin Neurosci.

[7] Pehler S-R, Craft-Rosenberg M (2009). Longing: the lived experience of spirituality in adolescents with Duchenne muscular dystrophy. J Pediatr Nurs.

[8] Spies S, Schipper K, Nollet F, Abma TA (2010). Duchenne muscular dystrophy. BMJ.

[9] Gibson BE, Zitzelsberger H, McKeever P (2009). ‘Futureless persons’: shifting life expectancies and the vicissitudes of progressive illness. Sociol Health Illn.

[10] Lindsay S, Cagliostro E, McAdam L (2019). Meaningful occupations of young adults with muscular dystrophy and other neuromuscular disorders. Can J Occup Ther.

[11] Gibson BE, Mistry B, Smith B, Yoshida KK, Abbott D, Lindsay S (2014). Becoming men: gender, disability, and transitioning to adulthood. Health.

[12] Schwartz CE, Stark RB, Audhya IF, Gooch KL (2021). Characterizing the impact of Duchenne Muscular Dystrophy on caregivers: a case-control investigation. J Patient-Rep Outcomes.

[13] Ciafaloni E, Fox DJ, Pandya S, Westfield CP, Puzhankara S, Romitti PA (2009). Delayed diagnosis in Duchenne Muscular Dystrophy: data from the Muscular Dystrophy Surveillance, tracking, and research network (MD STARnet). J Pediatr.

[14] Pane M, Lombardo ME, Alfieri P, D'Amico A, Bianco F, Vasco G (2012). Attention deficit hyperactivity disorder and cognitive function in Duchenne muscular dystrophy: phenotype-genotype correlation. J Pediatr.

[15] Sarepta Therapeutics (2020) Duchenne: a progressive, muscle-weakening disease. https://www.duchenne.com/disease-progression. Accessed March 3 2020

[16] Nereo NE, Hinton VJ (2003). Three wishes and psychological functioning in boys with Duchenne muscular dystrophy. J Dev Behav Pediatr: JDBP.

[17] Rapkin BD, Schwartz CE (2004). Toward a theoretical model of quality-of-life appraisal: implications of findings from studies of response shift. Health Qual Life Outcomes.

[18] Rapkin BD, Garcia I, Michael W, Zhang J, Schwartz CE (2016). Distinguishing appraisal and personality influences on quality of life in chronic illness: introducing the quality-of-life appraisal profile version 2. Qual Life Res.

[19] Schwartz CE, Stark RB, Rapkin BD (2020). Capturing patient experience: does quality-of-life appraisal entail a new class of measurement?. J Patient-Rep Outcomes.

[20] Schwartz CE, Stark RB, Rapkin BD (2021). Creating idiometric short-form measures of cognitive appraisal: balancing theory and pragmatics. J Patient-Rep Outcomes.

[21] Li Y, Rapkin BD (2009). Classification and regression tree analysis to identify complex cognitive paths underlying quality of life response shifts: a study of individuals living with HIV/AIDS. J Clin Epidemiol.

[22] Fleiss JL (1971). Measuring nominal scale agreement among many raters. Psychol Bull.

[23] Cohen J (1960). A coefficient of agreement for nominal scales. Educ Psychol Measur.

[24] Altman DG (1999). Practical statistics for medical research.

[25] Landis JR, Koch GG (1977). The measurement of observer agreement for categorical data. Biometrics.

[26] Miller GA, Chapman JP (2001). Misunderstanding analysis of covariance. J Abnormal Psych.

[27] Austin P (2011). An introduction to propensity score methods for reducing the effects of confounding in observational studies. Multivar Behav Res.

[28] Norman GR, Sloan JA, Wyrwich KW (2003). Interpretation of changes in health-related quality of life: the remarkable universality of half a standard deviation. Med Care.

[29] IBM (2020). IBM SPSS statistics for windows.

[30] R: A language and environment for statistical computing (2019) (R version 3.6.2 (2019-1-12) ed., pp. Open Source Statistical Software). Vienna, Austria: R Foundation for Statistical Computing

[31] Rijken M, Spreeuwenberg P, Schippers J, Groenewegen PP (2013). The importance of illness duration, age at diagnosis and the year of diagnosis for labour participation chances of people with chronic illness: results of a nationwide panel-study in the Netherlands. BMC Public Health.

[32] Needs BP and Education P. Aspirations index

[33] Deci EL and Ryan RM (2012) Self-determination theory

[34] Ntoumanis N, Ng JY, Prestwich A, Quested E, Hancox JE, Thøgersen-Ntoumani C (2021). A meta-analysis of self-determination theory-informed intervention studies in the health domain: effects on motivation, health behavior, physical, and psychological health. Health Psychol Rev.

[35] Pinquart M, Silbereisen RK, Fröhlich C (2009). Life goals and purpose in life in cancer patients. Support Care Cancer.

[36] Emmons RA, Keyes CLM, Haidt J (2003). Personal goals, life meaning, and virtue: wellsprings of a positive life. Flourishing: positive psychology and the life well-lived.

[37] Street H (2002). Exploring relationships between goal setting, goal pursuit and depression: a review. Aust Psychol.

[38] Rothbaum F, Morling B, Rusk N (2009). How goals and beliefs lead people into and out of depression. Rev Gen Psychol.

[39] Uttley L, Carlton J, Woods HB, Brazier J (2018). A review of quality of life themes in Duchenne muscular dystrophy for patients and carers. Health Qual Life Outcomes.

[40] Reilly MC, Lavin PT, Kahler KH, Pariser DM (2003). Validation of the Dermatology life quality index and the work productivity and activity impairment-chronic hand dermatitis questionnaire in chronic hand dermatitis. J Am Acad Dermatol.

[41] Davison G, Aldridge J, Manning S and Childs A (2011) A qualitative study exploring the impact on social well-being of young people living with a life-limiting neuromuscular disease: Poster 044. Dev Med Child Neurol 53

[42] Månsson R, Joffe MM, Sun W, Hennessy S (2007). On the estimation and use of propensity scores in case-control and case-cohort studies. Am J Epidemiol.

[43] Fairclough DL (2010) Simple imputation. In: Design and analysis of quality of life studies in clinical trials. Chapman and Hall/CRC, Boca Raton, FL, pp 164

[44] Cohen J (1992). A power primer. Psych Bull.

[45] Hanson C, Hirst W (1989). On the representation of events: a study of orientation, recall, and recognition. J Exp Psychol Gen.

[46] Tourangeau R, Rips LJ, Rasinski K (2000). The psychology of survey response.

